# Trends and inequalities in children aged 6–59 months who received Vitamin A supplementation: evidence from the 2003, 2008 and 2014 Ghana Demographic and Health Survey

**DOI:** 10.1186/s41182-022-00488-3

**Published:** 2022-12-28

**Authors:** Joshua Okyere, Simon Agongo Azure, Eugene Budu, Felix Mensah, Bright Opoku Ahinkorah, Edward Kwabena Ameyaw, Abdul-Aziz Seidu

**Affiliations:** 1 Department of Population and Health, University of Cape Coast, Cape Coast, Ghana; 2 Department of Nursing, College of Health Sciences, Kwame Nkrumah University of Science and Technology, Kumasi, Ghana; 3 Population and Reproductive Health Division, School of Public Health, University of Port Harcourt, Choba, Port Harcourt, Nigeria; 4 Department of Community Health, College of Health, Yamfo, Ghana; 5 Korle Bu Teaching Hospital, P.O. Box, 77, Accra, Ghana; 6 Department of Data Science and Economic Policy, University of Cape Coast, Cape Coast, Ghana; 7 REMS Consult Ltd., Sekondi-Takoradi, Western Region Ghana; 8 School of Public Health, Faculty of Health, University of Technology Sydney, Sydney, Australia; 9 Institute of Policy Studies and School of Graduate Studies, Lingnan University, Hong Kong, China; 10 L & E Research Consult Ltd., Wa, Upper West Region Ghana; 11 Centre for Gender and Advocacy, Takoradi Technical University, Takoradi, Ghana; 12 College of Public Health, Medical and Veterinary Sciences, James Cook University, Townsville, Australia

**Keywords:** Children, Ghana, Trends, Vitamin A supplementation, Demographic and Health Survey

## Abstract

**Background:**

Vitamin A deficiency is considered a public health issue, particularly among children under 5 years. Vitamin A supplementation is among the ten key essential nutrition actions put in place to tackle malnutrition in children and helps to reduce under-five mortality by almost a quarter in Vitamin A deficient areas. We, therefore, examined inequalities in Vitamin A uptake among children 6–59 months in Ghana.

**Methods:**

We used data from the 2003, 2008, and 2014 Ghana Demographic and Health Surveys. The WHO’s HEAT version 3.1 software was used for all the analyses. We adopted six equity stratifiers (maternal age, economic status, level of education, place of residence, sex of the child, and region) to disaggregate Vitamin A supplementation among children 6–59 months. Four measures were used to compute inequality, namely, Difference (D), Population Attributable Risk (PAR), Population Attributable Fraction (PAF) and Ratio (R).

**Results:**

Over the 11-year period, the proportion of children who received Vitamin A supplementation declined from 78.6% to 65.2%. There were inequalities by maternal age, particularly in 2003 (D = 13.1, CI: 2.3, 23.9; PAF = 0.5, CI: − 12.3, 13.2). The widest inequality in Vitamin A supplementation by economic status was noted in 2003 (D = 8.8, CI: 3.3–14.2; PAF = 8.3, CI: 5, 11.5). In terms of sex, the indices revealed mild inequality in Vitamin A supplementation throughout the period studied. For education, the highest inequality was observed in 2014 (D = 11.6, CI: 6.0, 17.1), while the highest inequality in terms of place of residence was observed in 2003 (D = 4.0, CI: − 0.1–8.4). In the case of region, substantial inequality was noted in 2014 (D = 34.7, CI: 22.6, 46.8; PAF = 21.1, CI: 15.3, 27).

**Conclusions:**

We conclude that there is a need for the government of Ghana to deploy targeted interventions to enhance the uptake of Vitamin A supplementation among the most disadvantaged subpopulations. Interventions targeted at these disadvantaged populations should be pro-poor in nature. In addition, the inequalities in the dimension of place of residence were mixed, favoring both rural and urban children at different points. This calls for a comprehensive and all-inclusive approach that enhances Vitamin A supplementation uptake in an equitable manner in both areas of residence. Empowerment of women through formal education could be an important step toward improving Vitamin A supplementation among children in Ghana.

## Introduction

In 2020, undernutrition contributed to 45% of child death worldwide [1]. Undernutrition refers to insufficient intake of energy and other nutrients to meet body requirements [2]. Vitamin A is one of such essential nutrients required for healthy vision and immunity [3]. Vitamin A deficiency is considered an issue of public health importance, particularly among children under 5 years. Vitamin A supplementation is among the ten key essential nutrition actions put in place to tackle malnutrition in children [4]. Furthermore, Vitamin A supplementation helps to reduce under-five mortality by almost a quarter in Vitamin A deficient areas [4]. WHO recommends that children should receive Vitamin A supplements twice a year and continue to receive this supplement up to 5 years of age, especially in areas, where Vitamin A is a public health problem [4]. Thus, children, 6–11 months receive 100,000 International Units (IU) and children 12–59 months receive 20,000 IU administered orally. A study by Berde et al. [5] in 23 sub-Saharan African countries revealed 59.4% of children 6–59 months had received Vitamin A supplementation.

There are inequalities that account for the uptake of Vitamin A supplementation. For example, in Nigeria, mothers’ education, residing in South–South region and having the highest wealth quintile were associated with higher odds of receiving Vitamin A supplementation [6]. In addition, in Bangladesh, children between 25 and 36 months, those whose mothers and fathers had attained higher education and those whose mothers had been exposed to mass media were more likely to consume Vitamin A supplements [7]. In Ghana, a study on Vitamin A supplementation among preschool children revealed that the nature of housing material, the ability of caretakers to correctly identify Vitamin A supplements and knowledge of the medical effects of Vitamin A deficiency influenced the uptake of Vitamin A supplementation [8].

Strategies such as community-directed treatments, distributor incentives, intensified education, and communication have been shown to improve coverage of Vitamin A supplementation [9]. Despite these strategies, there are limited studies on the inequalities of Vitamin A supplementation in Ghana. Hence, understanding this disparity will help to improve the health of children and promote the uptake of Vitamin A supplementation and by extension reduce morbidity and mortality among children under 5 years. Moreover, policy makers will find this study relevant in designing interventions that can optimize the coverage of Vitamin A uptake. This study seeks to examine inequalities in Vitamin A uptake among children 6–59 months in Ghana.

## Materials and methods

### Study design and data source

The study setting is Ghana. Data from the Ghana Demographic and Health Surveys (GDHS) from 2003, 2008, and 2014 were analyzed. GDHS forms part of the global surveys implemented by Measure Demographic and Health Surveys (DHS) in 85 low-and-middle-income countries (LMICs) worldwide. The overarching focus of DHS is to collate information on children, women and men. In the DHS, a cross-sectional design was used. DHS employs a two-stage cluster sampling technique to obtain a nationally representative data per place of residence and region. Selection of enumeration areas (EAs) is the first step and takes cognizance of rural and urban locations of Ghana. This is ensured by household selection from the EAs. The complete sampling procedure has been elaborated in the final reports of the 2003, 2008 and 2014 GDHS. The population for this study was mothers of children aged 6–59 months.

### Variables

The study's outcome was whether children 6–59 months have received Vitamin A supplements or not. Children who received Vitamin A supplements were categorised as “1”, while those who did not receive Vitamin A supplement were classified otherwise “0”. Six stratifiers were used to assess inequality in Vitamin A supplementation: mother’s age (15–19 years, 20–49 years), economic status measured by wealth quintile (quintile 1 [poorest], 2, 3, 4, 5 [richest]), level of education (no education, primary, secondary/higher), place of residence (rural, urban), sex of the child (male, female) and region (Western, Central, Greater Accra, Volta, Eastern, Ashanti, Brong Ahafo, Northern, Upper West, Upper East). Wealth quintile is derived by employing Principal Component Analysis (PCA). Level of education is measured by highest level of formal education completed [10].

### Statistical analyses

We used the 2019 updated WHO’s HEAT software, version 3.1, for all analyses [11]. Estimates and uncertainty intervals (UIs) of Vitamin A supplementation with respect to the aforementioned stratifiers were computed. Four measures were used to compute inequality, namely, Difference (D), Population Attributable Risk (PAR), Population Attributable Fraction (PAF) and Ratio (R). Two of these are simple unweighted measures (D, R) and two are complex weighted measures (PAR, PAF). At the same time, R and PAF are relative measures, whereas D and PAR are absolute measures. Summary measures were considered, because WHO [12] has indicated that both absolute and relative summary measures are essential for generating policy driven findings [11]. Unlike simple measures, the complex ones take size of categories inherent in a sub-population into account. WHO has extensively elaborated the procedure for generating summary measures [12, 13].

In calculating D in age, we deducted the prevalence of Vitamin A supplementation among children aged 6–59 months of mothers aged 15–19 years from the prevalence of those of mothers aged 20–49 years. For economic status, we deducted the prevalence of Vitamin A supplementation among children aged 6–59 months of mothers who are in the poorest wealth category from the prevalence of those of mothers who are in the richest group. For level of education, we computed D by deducting mothers of children 6–59 months who received Vitamin A supplementation and have had “no formal education” from mothers of children 6–59 months who received Vitamin A supplementation and had “secondary/higher education”, while D in place of residence was about inequality between rural and urban residents. For sex of the child, we calculated D by deducting male children 6–59 months who received Vitamin A supplementation from female children 6–59 months who received Vitamin A supplementation. With respect to region, D was computed by deducting the region with lowest estimate from the region with the highest estimate.

We computed the R for the variables with ordered responses such as level of education and wealth quintile as the difference between the most-disadvantaged sub-group (lowest quintile and uneducated) and the most-advantaged sub-group (highest quintile and secondary/higher). We derived PAR by computing the difference in Vitamin A supplementation estimate of the reference category (*yref*) and overall average of the prevalence of children 6–59 months who received Vitamin A supplementation. With respect to the ordered variables, *y*_*ref*_ referred to the most-advantaged subgroups. In the case of region, which was non-ordered, y_ref_ meant the region with the lowest estimate. The PAF was gauged by distributing PAR by the overall average μ, and further multiplied by 100 (PAF = [PAR / μ] × 100). Zero (0) PAF or PAR means no inequality, while a higher value indicates a relatively higher inequality. Variation in children 6–59 months who received Vitamin A supplement over the period was explored by making reference to the 95% UIs of the survey years. Absence of overlap in the UIs means that a statistically significant difference existed between the UIs and vice versa.

### Ethical approval

All GDHS survey data are freely available to the public. All surveys were approved by the Inner City Fund (ICF) International and the Ghana Health Service. The Measure DHS Program also ensured that the survey protocols complied with the U.S. Department of Health and Human Services regulations for protection of human subjects.

## Results

### Proportion of children aged 6–59 months who received Vitamin A supplementation by different inequality stratifiers, 2003, 2008 and 2014

As shown in Table 1, inequality in the receipt of Vitamin A supplementation between 2003 and 2014 was assessed with the following indicators: age, economic status, education, place of residence, sex and region. Over the 11-year period studied (2003–2014), the proportion of children who received Vitamin A supplementation declined from 78.6% in 2003 to 65.2% in 2014. Meanwhile, the least uptake occurred in 2008 (58.2%). The analysis showed that across ages, children of women in the 20–49 age range had the highest uptake in 2003 (78.9, CI: 76.8, 80.9) and 2008 (58.3, CI: 55.3, 61.3) but children of 15–19 aged women dominated in Vitamin A supplementation in the 2014 survey (66.7% CI: 54.3, 77.2).

**Table 1:** Proportion of children aged 6–59 months who received Vitamin A supplementation by inequality stratifiers, 2003, 2008, and 2014

Dimension	2003 (78.6%)		2008 (58.2%)		2014 (65.2%)	
	Sample	Proportion (%) [95% CI]	Sample	Proportion (%) [95% CI]	Sample	Proportion (%) [95% CI]
Mother’s age						
15–19 years	84	65.8 [54.5, 75.6]	82	55.6 [42.6, 67.8]	157	66.7 [54.3, 77.2]
20–49 years	2942	78.9 [76.8, 80.9]	2332	58.3 [55.3, 61.3]	4703	65.2 [62.3, 68]
Economic status						
Quintile 1 (poorest)	788	76.3 [72.7, 79.6]	621	51.3 [45.8, 56.7]	1057	59 [54.1, 63.8]
Quintile 2	658	75.6 [70.6, 80]	546	59.3 [54.1, 64.4]	1027	71.2 [67, 75.1]
Quintile 3	576	79.2 [73.9, 83.6]	441	57.6 [51.6, 63.3]	948	67.7[62.5, 72.5]
Quintile 4	530	79.2 [74.1, 83.5]	459	65.2 [59.3, 70.7]	920	61.9 [55.5, 67.9]
Quintile 5 (richest)	474	85.1 [80.3, 88.8]	347	60.6 [53.1, 67.5]	908	66.5 [62.1, 70.7]
Level of education						
No education	1200	74.6 [71.1, 77.7]	791	54 [49.2, 58.6]	1314	57.7 [53.4, 61.9]
Primary school	694	75.2 [70.4, 79.4]	590	57.9 [52.6, 63.1]	972	64.9 [60.2, 69.3]
Secondary/higher	1132	84.9 [82, 87.4]	1034	61.6 [57.5, 65.6]	2574	69.2 [65.6, 72.6]
Place of residence						
Rural	2009	77.2 [74.5, 79.7]	1509	57.9 [54, 61.6]	2658	67.7 [63.4, 71.8]
Urban	1017	81.2 [77.9, 84.2]	906	58.8 [54, 63.5]	2202	62.2 [58.6, 65.8]
Sex of the child						
Females	1511	77.4 [74.5, 80]	1171	56.4 [52.7, 63.5]	2332	65.1 [61.6, 68.5]
Males	1515	79.8 [77.1, 82.2]	1243	60 [56.2, 63.6]	2528	65.3 [62.2, 68.4]
Region						
Ashanti	563	82.7 [78, 86.6]	468	67.4 [60, 74]	902	64.2 [56.7, 71.1]
Brong Ahafo	328	75.1 [69.1, 80.3]	234	53.8 [45.4, 62]	443	78.2 [70.5, 84.4]
Central	259	66.8 [58.4, 74.2]	229	59.8 [50.7, 68.3]	538	79 [67.2, 87.4]
Eastern	313	78.4 [71, 84.3]	216	50.3 [42.5, 58.1]	449	71.4 [65.5, 76.7]
Greater Accra	339	75 [68.1, 80.9]	289	55.8 [46.4, 64.9]	769	54 [48.5, 59.4]
Northern	409	78.3 [73.6, 82.4]	357	42.3 [34.5, 50.4]	579	44.3 [37.8, 51.1]
Upper East	186	85.5 [73.4, 92.7]	125	68.1 [59.1, 75.9]	193	67.6 [60.8, 73.7]
Upper West	91	85.1 [76.8, 90.8]	63	69.9 [62.1, 76.8]	121	68.9 [60.2, 76.5]
Volta	242	82.2 [73.4, 88.5]	208	65.2 [55.8, 73.6]	372	76.9 [70.1, 82.5]
West	297	80.1 [73.1, 85.7]	225	62.7 [52.6, 71.8]	493	66.3 [60.9, 71.2]

In 2003 and 2014, receipt of Vitamin A supplementation was highly recorded among children from fifth (85.1% CI: 80.3, 88.8) and second (71.2% CI: 67, 75.1) quintile households correspondingly. Throughout the period studied, Vitamin A supplementation uptake favoured children whose mothers had secondary/higher education as evident in the 2014 survey (69.2% CI: 65.6, 72.6). Dominance in Vitamin A uptake occurred among urban women in 2003 (81.2% CI: 77.9, 84.2) but this was not the situation in 2014 as rural children led (67.7% CI: 63.4, 71.8). Male children exceeded the females in the uptake of Vitamin A supplementation in all the three surveys including the 2014 survey (65.3% CI 62.2, 68.4). Across the administrative regions, we found that almost nine out of ten children in the Upper East region received Vitamin supplementation (85.5% CI: 73.4, 92.7) and this was the highest in 2003. In 2014, children from Central Region overtook their Upper East counterparts with 79% (CI: 67.2, 87.4).

### Inequality indices of children aged 6–59 months who received Vitamin A supplementation, 2003, 2008 and 2014

In Table 2, findings on the inequality indices of estimates of factors associated with children aged 6–59 months who received Vitamin A supplementation are presented. These findings comprised simple (D, R) and complex (PAF and PAR) measures. There were inequalities by maternal age, particularly in 2003 in terms of simple (D = 13.1, CI: 2.3, 23.9) and complex measures (PAF = 0.5, CI: − 12.3, 13.2). The widest inequality in Vitamin A supplementation by economic status was noted in the 2003 survey and this manifested in the simple (D = 8.8, CI: 3.3–14.2) and complex (PAF = 8.3, CI: 5, 11.5) measures as well. The indices revealed mild inequality in Vitamin A supplementation by the sex of the children throughout the period studied. For instance, in 2014, the simple measures indicated mild inequalities (R = 1, CI: 0.9, 1.1), while both complex measures showed no inequality (PAF = 0, CI: − 2, 2; PAR = 0, CI: − 1.3, 1.3). For education, the highest inequality was observed in 2014 (D = 11.6, CI: 6.0, 17.1). The highest inequality in terms of place of residence was observed in 2003 (D = 4.0, CI: − 0.1–8.4). In the case on region, substantial inequality was noted in 2014 and this was evident in both the simple (D = 34.7, CI: 22.6, 46.8) and complex (PAF = 21.1, CI: 15.3, 27) measures.

**Table 2:** Inequality indices of children aged 6–59 months who received Vitamin A supplementation, 2003, 2008 and 2014

Dimension	2003			2008			2014		
	Estimate	Lower bound	Upper bound	Estimate	Lower bound	Upper bound	Estimate	Lower bound	Upper bound
Mother’s age									
Difference (D)	13.1	2.3	23.9	2.7	-10.5	15.9	-1.5	-13.4	10.4
Population Attributable Fraction (PAF)	0.5	-12.3	13.2	0.2	-18.1	18.4	0	-11.1	11.1
Population Attributable Risk (PAR)	0.4	-9.7	10.4	0.1	-10.5	10.7	0	-7.3	7.3
Ratio (R)	1.2	1	1.4	1	0.8	1.3	1	0.8	1.2
Economic status									
Difference (D)	8.8	3.3	14.2	9.3	0.3	18.3	7.5	1	14
Population Attributable Fraction (PAF)	8.3	5	11.5	4	− 1.8	9.8	2	− 2	6
Population Attributable Risk (PAR)	6.5	4	9	2.3	− 1	5.7	1.3	− 1.3	3.9
Ratio (R)	1.1	1	1.2	1.2	1	1.4	1.1	1	1.3
Level of education									
Difference (D)	10.4	6.1	14.6	7.7	1.5	13.8	11.6	6	17.1
Population Attributable Fraction (PAF)	8.1	5.7	10.4	5.8	1	10.7	6.1	2.7	9.6
Population Attributable Risk (PAR)	6.4	4.5	8.2	3.4	0.6	6.2	4	1.7	6.3
Ratio (R)	1.1	1.1	1.2	1.1	1	1.3	1.2	1.1	1.3
Place of residence									
Difference (D)	4	− 0.1	8.1	1	− 5.1	7.1	− 5.5	− 11	0
Population Attributable Fraction (PAF)	3.4	2.1	4.7	1	− 1.6	3.7	0	− 1.9	1.9
Population Attributable Risk (PAR)	2.7	1.6	3.7	0.6	− 0.9	2.1	0	− 1.2	1.2
Ratio (R)	1.1	1	1.1	1	0.9	1.1	0.9	0.8	1
Sex of the child									
Difference (D)	− 2.4	− 6.1	1.3	− 3.6	− 8.8	1.6	− 0.2	− 4.8	4.4
Population Attributable Fraction (PAF)	0	− 1.9	1.9	0	− 3.3	3.3	0	− 2	2
Population Attributable Risk (PAR)	0	− 1.5	1.5	0	− 1.9	1.9	0	− 1.3	1.3
Ratio (R)	1	0.9	1	0.9	0.9	1	1	0.9	1.1
Region									
Difference (D)	18.7	6.4	31	27.7	16.8	38.5	34.7	22.6	46.8
Population Attributable Fraction (PAF)	8.8	1.9	15.8	20.1	11.9	28.3	21.1	15.3	27
Population Attributable Risk (PAR)	6.9	1.5	12.4	11.7	7	16.5	13.8	10	17.6
Ratio (R)	1.3	1.1	1.5	1.7	1.3	2.1	1.8	1.5	2.2

## Discussion

The present study sought to examine the trends of inequalities of Vitamin A uptake among children 6–59 months in Ghana from 2003 to 2014 using five inequality dimensions. Our findings indicate that the prevalence of Vitamin A supplementation in 2003, 2008 and 2014 were 78.6%, 58.2% and 65.2%, respectively. The current proportion of Vitamin A supplementation in Ghana (65.2%) is lower than reports from other SSA countries, such as Namibia (83.6%), Rwanda (86.4%), Sierra Leone (83.2%), Senegal (88.4%), and Togo (81.7%) [5]. However, the result from this study is higher than reports from a related study conducted in Ethiopia [14].

We observed economic disparities in the trends of Vitamin A supplementation among children in Ghana. Consistently across the survey periods, our study revealed that children in higher wealth quintiles reported higher proportion of Vitamin A supplementation. Similar findings have been reported in Ethiopia [14] and India [15]. A plausible explanation for this finding could be that parents in higher wealth quintile households are more likely to have easy access to healthcare facilities [16]. Despite the existence of the free maternal health care policy and national health insurance scheme (NHIS) which seek to offset out-of-of-pocket payment for health services including Vitamin A supplementation, there are latent expenses such as cost of transportation that are not covered by these policies [17, 18]. Therefore, children born to mothers in poorer wealth quintiles become significantly disadvantaged as their mothers may struggle to afford these latent expenses. Our absolute and relative indices revealed that the widest inequality by wealth was reported in 2003. This finding is not surprising because prior to 2003, Ghana had not implemented the NHIS and free maternal health policies [18, 19]. Hence, women were paying out-of-pocket for postnatal care services and latent health expenditures.

Our study also revealed the existence of inequalities in Vitamin A supplementation across the dimension of education. Vitamin A supplementation was consistently high among children born to mothers with secondary or higher education across the various survey points. This finding mirrors results from previous studies that have found high Vitamin A supplementation among children whose mothers have secondary or higher education [14, 15]. The result from this study may be explained from the perspective that formal education and higher education mothers’ awareness of health and nutritional requirements contribute positively toward Vitamin A supplementation [5]. Our indices estimate shows that the widest inequality in Vitamin A supplementation by mothers’ education was reported in 2014. We are unable to postulate plausible explanations for the wide inequality in 2014 across the dimension of education.

We found residence-based inequalities in the uptake of Vitamin A supplements. Similar findings have been reported in related studies that showed that there are rural–urban inequalities in the uptake of Vitamin A supplements in Ethiopia [14], India [15], and SSA [5]. In this study, the proportion of Vitamin A supplementation was high among children born to urban dwelling women in 2003 and 2008. However, in 2014, the widest inequality in Vitamin A supplementation favored children born to rural dwelling women. Perhaps, the changing dynamics in the uptake of Vitamin A supplementation in Ghana could be a reflection of the outcome of several pro-rural maternal and child health policies or interventions, such as the Community-Based Health Planning and Services (CHPS), free maternal healthcare policy and NHIS [20, 21]. The findings imply that inasmuch as pro-rural interventions are critical to the uptake of Vitamin A supplementation, there is a need to focus on children born to urban dwelling women. This is important to reduce substantial rural–urban inequalities.

Consistently across the various survey points, more males received Vitamin A supplements compared to female children. This result is similar to the findings of a study conducted in Nigeria that revealed that males dominate the uptake of Vitamin A supplementation [22]. Adamu and Muhammed [22] posit that more males receive Vitamin A supplementation possibly because males tend to have high enrollment in school hence they have greater access to Vitamin A supplementation. Notwithstanding, the simple inequality estimates showed mild inequalities, whereas the complex indices revealed no inequality across the dimension of sex. This is consistent with previous studies that have found no inequalities in Vitamin A supplementation across the dimension of sex [5, 6, 23, 24].

There were mixed inequalities in the uptake of Vitamin A supplementation across the dimension of age. In 2003 and 2008, children born to mothers of older age (20–49 years) had the highest Vitamin A supplementation uptake. The findings mirror results from previous studies conducted in SSA [5] and Nigeria [25]. A plausible explanation could be that, women who deliver after the age of 20 or later tend to have a better understanding of the nutritional and health requirements of their children [26]. Another possible justification for this could be that, women aged 15–19 years are in the category of adolescence. Traditionally in Ghana, adolescents are not expected to bear children [27]. As such, there is a lot stigmatization attached to adolescent motherhood which tends to reduce their health-seeking behavior.

In 2014, children born to young women (15–19 years) constituted the largest proportion of Vitamin A supplementation. The widest inequality was reported in 2003. Perhaps, this may be a reflection of the low priority for adolescent-and-youth-friendly services to young mothers. The study also revealed the existence of subnational regional inequalities in the uptake of Vitamin A supplementation in Ghana. This aligns with results from previous studies that found subnational regional disparities in the uptake of Vitamin A supplementation among children [14]. A plausible explanation for this could be the unequal distribution of healthcare services across the country makes accessibility to healthcare services difficult for some regions while being easy for other regions.

### Strengths and limitations

The study relied on nationally representative data (GDHS). Therefore, our findings are generalizable to the wider population of children aged between 6 and 59 months in Ghana. In addition, the study follows standardized analytical approaches which included using both absolute and relative indices to estimate the magnitude of inequality across the various dimensions. Nonetheless, there are some limitations that should be considered. We were limited to including only equity stratifiers that are available in the WHO health equity monitor database. Hence, other dimensions such as religion, ethnicity and parity could not be assessed. In addition, the results are based entirely on cross-sectional data; therefore, no causal association can be inferred.

## Conclusions

Vitamin A supplementation in Ghana has declined from 78.6% in 2003 to 58.2% in 2008 to 65.2% in 2014. The present study shows that there are inequalities in the uptake of Vitamin A supplementation among children 6–59 months in Ghana. These inequalities were visible across the dimensions of mother’s age, place of residence, wealth quintile, sex of the child, level of education, and region. Children from poorer wealth quintile households, those born to younger mothers, rural dwelling mothers and mothers with lower educational attainment were the most disadvantaged. There is a need for the government of Ghana and its partners to focus on targeted interventions to enhance the uptake of Vitamin A supplementation among the most disadvantaged subpopulations. Interventions targeted at these disadvantaged populations should be pro-poor in nature. In addition, the inequalities in the dimension of place of residence were mixed favoring both rural and urban children at different points. This calls for a comprehensive and all-inclusive approach that enhances Vitamin A supplementation in an equitable manner in both areas of residence. Empowerment of women through formal education could be another important step toward improving Vitamin A supplementation among children in Ghana.

## Data Availability

The data sets generated and/or analyzed during the current study are available in the WHO’s HEAT version 3.1 [https://www.who.int/gho/health_equity/assessment_toolkit/en/].

## Abbreviations

EAs: Enumeration areas
DHS: Demographic and Health Surveys
GDHS: Ghana Demographic and Health Surveys
PAF: Population Attributable Fraction
PAR: Population Attributable Risk
PCA: Principal Component Analysis
SDG: Sustainable Development Goals
SSA: Sub-Saharan Africa
